# Supplementary material for: Physiological and Proteomic Responses of Cassava to Short-Term Extreme Cool and Hot Temperature
Source: Plants (Basel). 2022 Sep 3;11(17):2307. doi: 10.3390/plants11172307 (PMC9460903; doi:10.3390/plants11172307)
Supplement: Supplementary file 1 [file plants-11-02307-s001.zip › plants-1880128-supplementary.pdf]

Supplementary Tables

Physiological and Proteomic Responses of Cassava to Short-term Extreme Cool and Hot Temperature

Supranee Santanoo, Kochaphan Vongcharoen , Poramate Banterng , Nimitr Vorasoot , Sanun Jogloy , Sittiruk Roytrakul, and Piyada Theerakulpisut*

Supplementary Table S1. The effects of short-term exposure to different temperatures (15, 30 and 45 °C) on net photosynthesis (P_n), stomatal conductance (gs) , transpiration rate (T_r) , water use efficency (WUE), intercellular CO_2 concentration (C_i), and ratio between P_n and C_i, maximum quantum yield efficiency of photosystem II (Fv'/Fm'), effective quantum yield of PSII photochemistry (ΦPSII), and electron transport rate (ETR), electrolyte leakage (EL), proline and malondialdehyde (MDA) of cassava cvs. Rayong 9 and Kasetsart 50. Means ± SD (n=6) which are significantly different ($p < 0.05$) among treatments for each cultivar are denoted by different lower-case letters, whereas those among temperatures across cultivar are represented by different capital letters. The significant differences between cassava genotypes are denoted with*.

Parameter	Genotype	Temperature			Critical-*p* value
		**15 °C**	**30 °C**	**45 °C**	
**P_n**	Rayong 9	6.07 ± 1.28 b	15.29 ± 1.79 a	8.60 ± 3.48 b	$p < 0.05$
(μmol CO_2 m^{-2} s^{-1})	Kasetsart 50	5.74 ± 3.95 b	15.56 ± 1.90 a	7.07 ± 1.49 b	$p < 0.05$
	**mean**	**5.91 B**	**15.43 A**	**7.84 B**	$p < 0.05$
**gs**	Rayong 9	0.07 ± 0.01 b	0.08 ± 0.02 b	0.24 ± 0.04 a	$p < 0.05$
(mol H_2O m^{-2} s^{-1})	Kasetsart 50	0.06 ± 0.04 b	0.10 ± 0.003 b*	0.23 ± 0.12 a	$p < 0.05$
	**mean**	**0.065 B**	**0.09 B**	**0.235 A**	$p < 0.05$
**T_r**	Rayong 9	2.31 ± 0.31 b	3.04 ± 0.56 b	13.37 ± 2.08 a	$p < 0.05$
(mmol H_2O m^{-2} s^{-1})	Kasetsart 50	2.26 ± 1.16 b	3.44 ± 0.69 b	12.44 ± 6.07 a	$p < 0.05$
	**mean**	**2.28 B**	**3.24 B**	**12.90 A**	$p < 0.05$
**WUE**	Rayong 9	2.63 ± 0.42 b	5.09 ± 0.56 a	0.64 ± 0.22 c	$p < 0.05$
(μmol CO_2 mmol H_2O^{-1})	Kasetsart 50	3.25 ± 1.29 b	4.62 ± 0.67 a	0.52 ± 0.14 c	$p < 0.05$
	**mean**	**2.94 B**	**4.85 A**	**0.58 C**	$p < 0.05$
**C_i**	Rayong 9	249 ± 24 b	70 ± 27 c	306 ± 18 a	$p < 0.05$
μmol CO_2 mol air^{-1}	Kasetsart 50	218 ± 62 b	124 ± 12 c*	312 ± 17 a	$p < 0.05$
	**mean**	**233.5 B**	**97 C**	**309 A**	$p < 0.05$
**P_n/C_i**	Rayong 9	0.02 ± 0.01 b	0.24 ± 0.09 a*	0.03 ± 0.01 b	$p < 0.05$
	Kasetsart 50	0.03 ± 0.02 b	0.13 ± 0.02 a	0.02 ± 0.01 b	$p < 0.05$
	**mean**	**0.03 B**	**0.19 A**	**0.03 B**	$p < 0.05$
**Fv'/Fm'**	Rayong 9	0.43 ± 0.04 b	0.50 ± 0.03 a	0.46 ± 0.03 ab	$p < 0.05$
	Kasetsart 50	0.43 ± 0.02 b	0.49 ± 0.02 a	0.47 ± 0.03 a	$p < 0.05$
	**mean**	**0.43 B**	**0.495 A**	**0.465 A**	$p < 0.05$
**ΦPSII**	Rayong 9	0.12 ± 0.01 b	0.18 ± 0.01 a	0.05 ± 0.02 c	$p < 0.05$
	Kasetsart 50	0.12 ± 0.02 b	0.19 ± 0.01 a	0.06 ± 0.03 c	$p < 0.05$
	**mean**	**0.12 B**	**0.185 A**	**0.055 C**	$p < 0.05$
**ETR**	Rayong 9	80.85 ± 5.69 b	119.36 ± 5.84 a	33.96 ± 10.44 c	$p < 0.05$
(μmol (e^-) m^{-2} s^{-1})	Kasetsart 50	83.40 ± 11.61 b	122.30 ± 6.41 a	37.01 ± 18.91 c	$p < 0.05$
	**mean**	**82.125 B**	**120.83 A**	**35.48 C**	$p < 0.05$
**Electrolyte leakage**	Rayong 9	74 ± 28 *	57 ± 21	47 ± 24	ns
(%)	Kasetsart 50	53 ± 30	58 ± 14	47 ± 17	ns

	**mean**	**63.5**	**57.5**	**47**	ns
**Proline**	Rayong 9	1.79 ± 0.32 c	6.84 ± 1.12 a*	5.55 ± 1.11b*	$p < 0.05$
(µg g^{-1} FW)	Kasetsart 50	3.28 ± 0.74*	3.54 ± 0.27	2.86 ± 0.90	ns
	**mean**	**2.54 B**	**5.19 A**	**4.21 AB**	$p < 0.05$
**Malondialdehyde**	Rayong 9	9.46 ± 1.45 a	6.24 ± 0.67 b	7.20 ± 0.63 b	$p < 0.05$
(nmol g^{-1} FW)	Kasetsart 50	12.04 ± 1.45 a*	7.53 ± 0.78 b*	7.74 ± 0.58 b	$p < 0.05$
	**mean**	**10.75 A**	**6.89 B**	**7.47 B**	$p < 0.05$

S**upplementary Table S2**. The quantitation of differentially expressed proteins in leaves of cassava cvs. Rayong 9 and Kasetsart 50 exposed to low (15 °C) and high temperature (45 °C) stress compared to those at normal temperature (30 °C).

**Protein ID**	**Gene names**	**Protein names**	**Mean quantitation of protein based on the peptide ion signal intensities of acquired LC-MS raw data (log2 value)**					
			**RY9 15 °C**	**KU50 15 °C**	**RY9 30 °C**	**KU50 30 °C**	**RY9 45 °C**	**KU50 45 °C**
A0A2C9V0E9	MANES_11G062900	AP2/ERF domain-containing protein	18.20	15.72	0.00	0.00	0.00	0.00
A0A2C9UPP7	MANES_13G007200	Glutaredoxin domain-containing protein	17.87	18.38	0.00	0.00	0.00	0.00
A0A2C9WDC0	MANES_02G127400	Thymidine kinase (EC 2.7.1.21)	16.51	0.00	0.00	0.00	0.00	0.00
A0A2C9VTU6	MANES_05G063700	Uncharacterized protein	16.92	0.00	0.00	0.00	0.00	0.00
A0A2C9UA68	MANES_16G085500	Cytokinin dehydrogenase (EC 1.5.99.12)	18.08	0.00	0.00	0.00	0.00	0.00
A0A2C9VH14	MANES_08G167700	Uncharacterized protein	0.00	21.35	0.00	0.00	0.00	0.00
A0A0C5A1R1	Ann2	Annexin	0.00	0.00	0.00	0.00	13.33	15.08
A0A2C9U0M7	MANES_18G047200	Uncharacterized protein	0.00	0.00	0.00	0.00	0.00	15.64
